# (Pro)renin Receptor Mediates Both Angiotensin II-Dependent and -Independent Oxidative Stress in Neuronal Cells

**DOI:** 10.1371/journal.pone.0058339

**Published:** 2013-03-14

**Authors:** Hua Peng, Wencheng Li, Dale M. Seth, Anand R. Nair, Joseph Francis, Yumei Feng

**Affiliations:** 1 Department of Physiology, Tulane Hypertension and Renal Center of Excellence, Tulane University School of Medicine, New Orleans, Louisiana, United States of America; 2 Department of Comparative Biomedical Sciences, School of Veterinary Medicine, Louisiana State University, Baton Rouge, Louisiana, United States of America; Chang Gung University, Taiwan

## Abstract

The binding of renin or prorenin to the (pro)renin receptor (PRR) promotes angiotensin (Ang) II formation and mediates Ang II-independent signaling pathways. In the central nervous system (CNS), Ang II regulates blood pressure via inducing oxidative stress; however, the role of PRR-mediated Ang II-independent signaling pathways in oxidative stress in the CNS remains undefined. To address this question, Neuro-2A cells were infected with control virus or an adeno-associated virus encoding the human PRR. Human PRR over-expression alone increased ROS levels, NADPH oxidase activity, as well as NADPH oxidase (NOX) isoforms 2 and 4 mRNA expression levels and these effects were not blocked by losartan. Moreover, the increase in NOX 2 and NOX 4 mRNA levels, NADPH oxidase activity, and ROS levels induced by PRR over-expression was prevented by mitogen activated protein kinase/extracellular signal-regulated kinase 1 and 2 (MAPK/ERK1/2) inhibition, and phosphoinositide 3 kinase/Akt (IP3/Akt) inhibition, indicating that PRR regulates NOX activity and ROS formation in neuro-2A cells through Ang II-independent ERK1/2 and IP3/Akt activation. Interestingly, at a concentration of 2 nM or higher, prorenin promoted Ang II formation, and thus further increased the ROS levels in cultured Neuro-2A cells via PRR. In conclusion, human PRR over-expression induced ROS production through both angiotensin II-dependent and -independent mechanisms. We showed that PRR-mediated angiotensin II-independent ROS formation is associated with activation of the MAPK/ERK1/2 and PI3/Akt signaling pathways and up-regulation of mRNA level of NOX 2 and NOX4 isoforms in neuronal cells.

## Introduction

It is well known that reactive oxygen species (ROS) play a pathophysiological role in the development of hypertension [1]–[4] and an activation of the renin-angiotensin system (RAS) is one of the key mediators in ROS production [5]. The (pro)renin receptor (PRR), a newly discovered member of the RAS, has been identified in various tissues including the brain, heart, placenta, liver, pancreas, lung, and kidney [6]. The binding of prorenin to PRR induces a conformational change of prorenin, conferring catalytic activity to prorenin, which leads to increased angiotensin II (Ang II) formation both *in vitro* and *in vivo* [7], [8]. In the CNS, Ang II has been shown to induce ROS production and activate neuronal excitability [9], [10]. In addition to promoting Ang II formation and activating signals downstream, the binding of renin or prorenin to the PRR activates several Ang II-independent intracellular signaling pathways, increasing production of profibrotic and proinflammatory factors as well as cellular proliferation [11], [12]. However, whether and how the PRR affects ROS production in neurons has not been defined. We hypothesized that the PRR mediates Ang II-dependent and -independent ROS formation in neuronal cells.

The present study shows that human PRR over-expression mediates both Ang II-dependent and -independent ROS formation and NADPH oxidase (NOX) activation in Neuro-2A cells. The Ang II-independent ROS formation and NOX activation induced by PRR over-expression is associated with phosphorylation of MAPK/ERK1/2 and phosphoinositide 3 kinase/Akt (PI3K/Akt). Our results provide an alternative mechanism by which the PRR influences oxidative stress in neuronal cells.

## Materials and Methods

### Ethics Statement

All procedures were conducted in accordance with the National Institutes of Health Guide for Care and Use of Laboratory Animals and were approved by the Institutional Animal Care and Use Committees at Tulane University School of Medicine.

### Adeno-associated Virus Generation

The adeno-associated virus (AAV) serotype 2 coding for human PRR (AAV-hPRR-eGFP) was developed in collaboration with the University of Iowa Gene Transfer Vector Core. The human PRR cDNA was a gift from Dr. Genevieve Nguyen at College de France. The plasmid AAV-hPRR-eGFP was digested with XbaI and SpeI to excise the 1061 bp human PRR fragment with a GFP tag. To ensure the natural structure of PRR, we designed and inserted an internal ribosome entry site sequence between the PRR and GFP gene. Thus, the PRR and GFP protein are expressed as two separate proteins instead of a fusion protein. The resulting construct was then used to generate the hPRR-eGFP adeno-associated virus as described [13].

### Cell Culture and AAV Infection

Mouse Neuro-2A cells (ATCC Manassas, VA) were infected with either AAV-hPRR-eGFP (10^−5^v.g./cell) virus or AAV-eGFP as controls (10^−5^v.g./cell) for 72 h. Cells were then starved overnight (serum free medium) followed by treatment with vehicle (PBS), losartan, mouse prorenin, wortmannin, U0126, or a combination for 30 min. At the end of the experiment, cells were collected for further analysis including dihydroethidium (DHE) staining, NADPH oxidase activity assay, real time PCR, and western blotting.

### Western Blot Analysis

Neuro-2A cells were harvested and lysed. Protein lysates (30 µg) were used for SDS-PAGE and blotting. Quantification was performed using NIH Image J software (http://rsbweb.nih.gov/ij/) in a blinded manner. The expression levels of targeted proteins were normalized based on the expression levels of β-actin protein.

### Measurement of ROS

ROS levels were measured using the oxidant-sensitive fluoroprobe DHE (Sigma-Aldrich, St. Louis, MO). Cells were incubated with DHE (5 µM) for 30 min. Images were captured (EVOS Digital Inverted Microscope) at Excitation/Emission wavelength (518/605 nm), and fluorescence intensity was quantified using Image J analysis software in a blinded manner.

### Measurement of Superoxide Using Electron Paramagnetic Resonance (EPR)

Free radical production rates in the Neuro2A cells were measured using EPR as described previously [14], [15]. All EPR measurements were performed using an EMX EPR eScan BenchTop spectrometer with a super-high quality factor (Q) microwave cavity (Bruker Company, Germany). For superoxide production, the Neuro2A cells were incubated with PEG-SOD (50 U/ml, Sigma) at 37°C for 30 min, and the spin probe CMH (200 µM) was added for another 30 min incubation period. The values obtained from incubation with PEG-SOD and CMH were subtracted from the values obtained from incubation with CMH only.

### Measurement of NADPH Oxidase Activity

NADPH oxidase activity was measured in cell lysate using a lucigenin luminescence assay. Chemiluminescence readings were obtained at 60 sec intervals for an overall measuring time of 60 min using a microplate luminometer (Mation Fluostar Optima Plate Reader). The relative luminescence units (RLU) were normalized to the cell amount.

### Ang II Measurement

Following a 72 h AAV-PRR-eGFP or control virus infection, cells were rinsed and changed to fresh serum-free media, and then incubated with vehicle or a different concentration of prorenin (0.1, 1, 2, 10 nM) for 20 min with or without pre-incubation with captopril (10 µM) for 30 min. Methanol denatured cell lysates were subjected to solid phase extraction kit (Aglient Tech, CA) and Ang II levels were measured using an Ang II Fluorescent Immunoassay EIA kit (Phoenix Pharmaceuticals, AZ).

### Measurement of Total Prorenin and Renin Level

Neuro-2A cell lysate and culture media were harvested three days after incubation in serum-free medium. Total (pro)renin (prorenin and renin) concentrations were measured using a mouse (pro)renin antigen assay kit (Molecular Innovations, Novi, MI) according to the manufacturer’s instructions.

### siRNA Transfection

The NOX2 (Sense: GGUCUUAUUUUGAAGUGUUTT, Anti-sense: AACACUUCAAAAUAAGACCTC), NOX4 (Sense: GAAAUUACCCUAAGUUAUATT, Anti-sense; UAUAACUUAGGGUAAUUUCTA), and scramble siRNA (Sense: GCTGTGATAAUCACCUCUAC, Anti-Sense: GATTGAGGUGAUUAUCACAGC) were designed and purchased from Ambion (Carlsbad, CA). Transfections were performed using Lipofectamine 2000 (Invitrogen,CA) according to the manufacturer’s instructions. Post transfection (48 h), cells were harvested for NOX2 and NOX4 real time PCR or used for DHE staining.

### RNA Isolation and Real-time PCR

Total RNA was isolated from Neuro-2A cells using an RNeasy kit (Qiagen Technologies, Germany). Specific primers for human PRR, mouse PRR, NOX1, NOX2, NOX4 and mouse glyceraldehyde 3-phosphate dehydrogenase (GAPDH) were designed using PrimerQuest Software (Integrated DNA Technologies, Coralville, IA). The expression levels of targeted mRNAs were normalized based on the expression levels of the GAPDH, used as an internal control, mRNA [16].

### Animal Study

Twelve-week-old, male, C57BL/6J mice were obtained from The Jackson Laboratory. Mice were intracerebroventricularly (ICV) injected with 100 nl of AAV-hPRR-eGFP or AAV-GFP (1.1×10^12^ vg/ml) as describe previously [17]. After 72 h, brain tissue from the subfornical organ (SFO) and cortex (as controls) were micro-punched using a cryostat and total RNA was isolated for measurement of NOX2, NOX4, and PRR mRNA levels using real time PCR.

### Statistical Analysis

All data were collected from three independent experiments and each experiment was performed in triplicate. Data, expressed as mean ±SEM, were analyzed by Student’s t test (2 groups) or two way ANOVA (multiple groups) followed by a Bonferroni post-hoc test to compare replicate means when appropriate. Statistical comparison was performed using Prism5 (GraphPad Software). Differences were considered statistically significant at P<0.05.

## Results

### Characterization of the Adeno-associated Virus Over-expressing PRR

**Figure 1:**
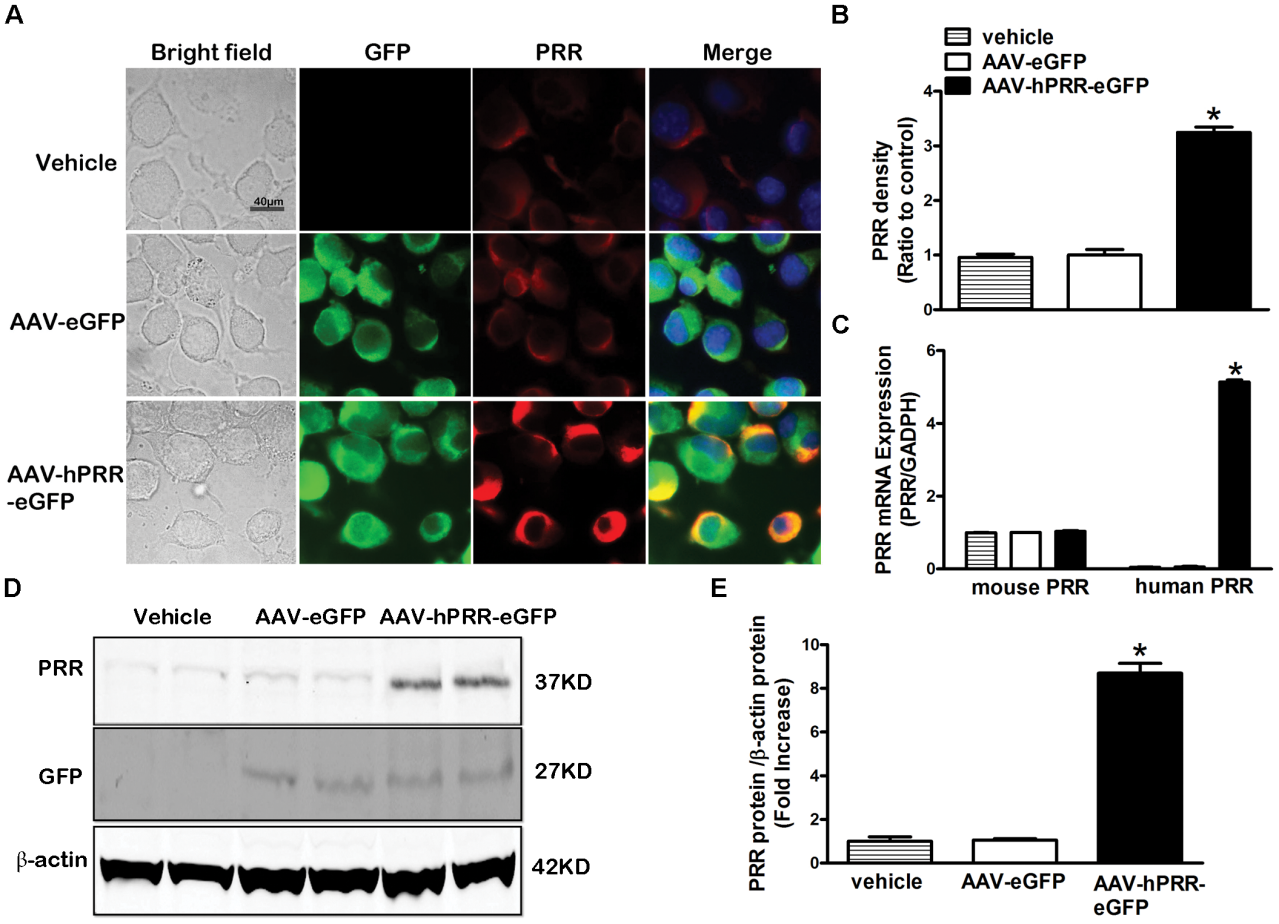
Characterization of AAV-hPRR-eGFP in Neuro-2A cells. Representative images (A) showing the GFP fluorescence (green), PRR immunofluorescence staining (red), nuclear staining by DAPI (blue), and the co-localization of GFP with PRR 3 d after virus infection with AAV-eGFP or AAV-hPRR-eGFP. A semi-quantitative graph of PRR immunostaining (B), a quantitative real-time PCR for mouse PRR and human PRR mRNA expression (C), and PRR and GFP protein expression by western blot (D) in Neuro-2A cells. *P<0.05 vs. AAV-eGFP.

We recently reported an increase in brain PRR levels in hypertensive mice where brain-targeted PRR knockdown attenuates hypertension [17]. To dissect the molecular mechanisms of the increased brain PRR in the development of hypertension, we developed a new AAV coding for human PRR upstream of an eGFP reporter gene. The AAV induced strong GFP fluorescence 3 days post infection of the Neuro-2A cells suggesting that GFP is a reliable index for AAV infection efficiency (Figure 1A). In cells infected with AAV-hPRR-eGFP, the PRR level was significantly increased compared with the AAV-eGFP infected cells and control cells (Figure 1B). In addition, AAV-hPRR-eGFP induced a significant increase in human PRR mRNA (Figure 1C, P<0.05) and protein levels (Figure 1D, E) in neuro-2A cells when compared with endogenous mouse PRR. These data suggest that our newly developed AAV-hPRR-eGFP can successfully infect and express human PRR in Neuro-2A cells.

### Human PRR Over-expression Induces both Ang II-dependent and -independent ROS Production in Neuro-2A Cells

**Figure 2:**
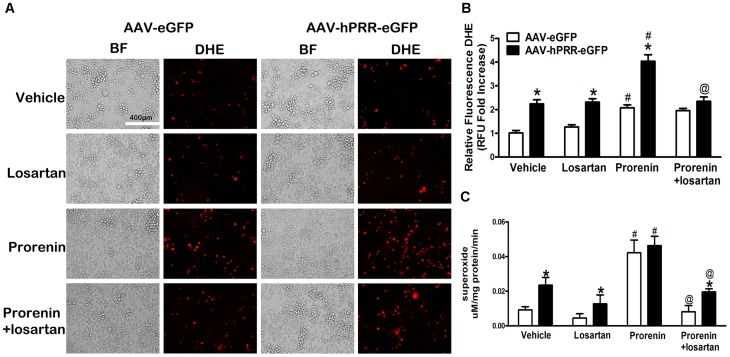
PRR over-expression induces Ang II-dependent and -independent ROS production in neuronal cells. Representative DHE staining images (A) and a semi-quantitative graph (B) for ROS levels 3 days post virus infection in Neuro-2A cells. Superoxide production (C) was measured by Electron paramagnetic resonance (EPR). * P<0.05 vs. AAV-eGFP with the same treatment, # P<0.05 vs. AAV-eGFP with vehicle. @ P<0.05vs. AAV-hPRR-eGFP with prorenin.

To examine whether the PRR participates in regulating oxidative stress in neuronal cells, total ROS and superoxide levels were measured using DHE staining and EPR. The PRR over-expression alone significantly increased ROS and superoxide levels compared with controls (Figure 2A, top two panels Figure 2B, C) and the increase in ROS and superoxide levels was not reversed by the Ang II type 1 receptor (AT1R) blocker, losartan, even at a concentration of 100 µm (Figure S1), indicating that human PRR over-expression induces Ang II-independent ROS formation. The addition of exogenous mouse prorenin (2 nM), a ligand for PRR, to the cell culture media, induced a significant increase in DHE fluorescence and superoxide levels compared with vehicle in both AAV-hPRR-eGFP and AAV-eGFP infected cells (Figure 2A, bottom two panels, P<0.01). Moreover, in the presence of exogenous prorenin, the total ROS levels, were significantly higher in cells over-expressing human PRR compared with cells infected with control virus (P<0.0001). Interestingly, losartan significantly decreased the DHE fluorescence and superoxide levels induced by the addition of exogenous prorenin (P<0.0001) indicating that 2 nM of prorenin mediates Ang II/AT1R-dependent ROS formation. However, the superoxide levels remained significantly higher in cells with PRR over-expression. Our results indicate that PRR over-expression increased ROS and superoxide production through both Ang II-dependent and -independent pathways.

### Ang II, Prorenin, and Renin Levels in Neuronal Cells

**Figure 3:**
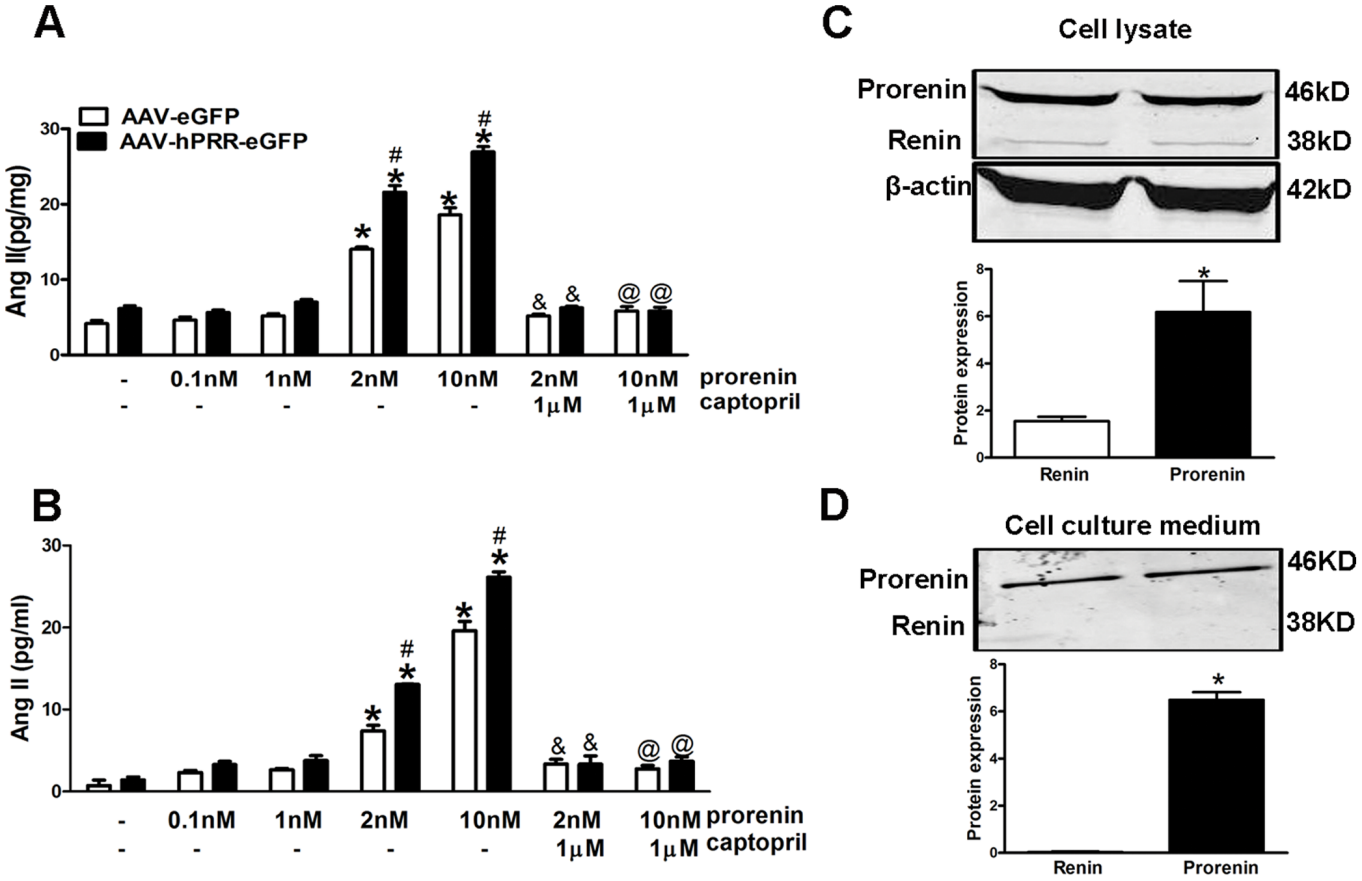
Ang II, prorenin, and renin levels in Neuro-2A cells. Ang II levels in cell lysate (A) and cell culture medium (B) following treatment of vehicle or mouse prorenin (0.1, 1, 2, and 10 nM; 20 min), with or without captopril (10 µM; 30 min) 3 d after virus infection. Representative western blotting and semi-quantitative graph of prorenin and renin protein levels in cell lysate (C) and cell culture medium (D). For panel A and B: *P<0.05 vs. AAV-eGFP with vehicle. # P<0.05 vs. AAV-eGFP with the same treatment. & P<0.05 vs. prorenin (2 nM). @ P<0.05 vs. prorenin (10 nM). For panel C and D: * P<0.05 vs. renin.

We measured the Ang II levels in cell lysate and culture medium under at different concentrations of prorenin to show that PRR mediates Ang II formation in Neuro-2A cells depending on prorenin levels. PRR over-expression alone did not significantly alter Ang II levels in the cell lysate or culture media following vehicle, 0.1 nM, or 1 nM of prorenin treatment (Figure 3A, B). However, the addition of 2 nM or 10 nM of prorenin induced a significant elevation of Ang II levels in both the neuro-2A cell lysate and culture medium (Figure 3A, B; P<0.01). The formation of Ang II was inhibited in the presence of an ACE inhibitor (captopril, 10 µM) confirming the derivation of Ang II from Ang I. To show the existence of endogenous renin and prorenin in Neuro-2A cells, we measured (pro)renin levels in both the cell lysate and culture medium. In Neuro-2A cells, both the cell lysate (17.7±0.2 ng/mg) and culture medium (54.3±2.0 ng/ml) contained significant amounts of total (pro)renin three d after incubation in serum-free medium. We performed western blotting for prorenin (46 kD) and renin (38 kD) to examine their proportion in the total (pro)renin. The prorenin levels were approximately 10-fold higher than those of renin in Neuro-2A cell lysates (Figure 3C, P<0.05), however prorenin was the major form of total (pro)renin in the cell culture medium, while renin was almost undetectable by western blotting (Figure 3D).

### PRR Mediates ROS Production via Up-regulation of NOX2 and NOX4 Levels

**Figure 4:**
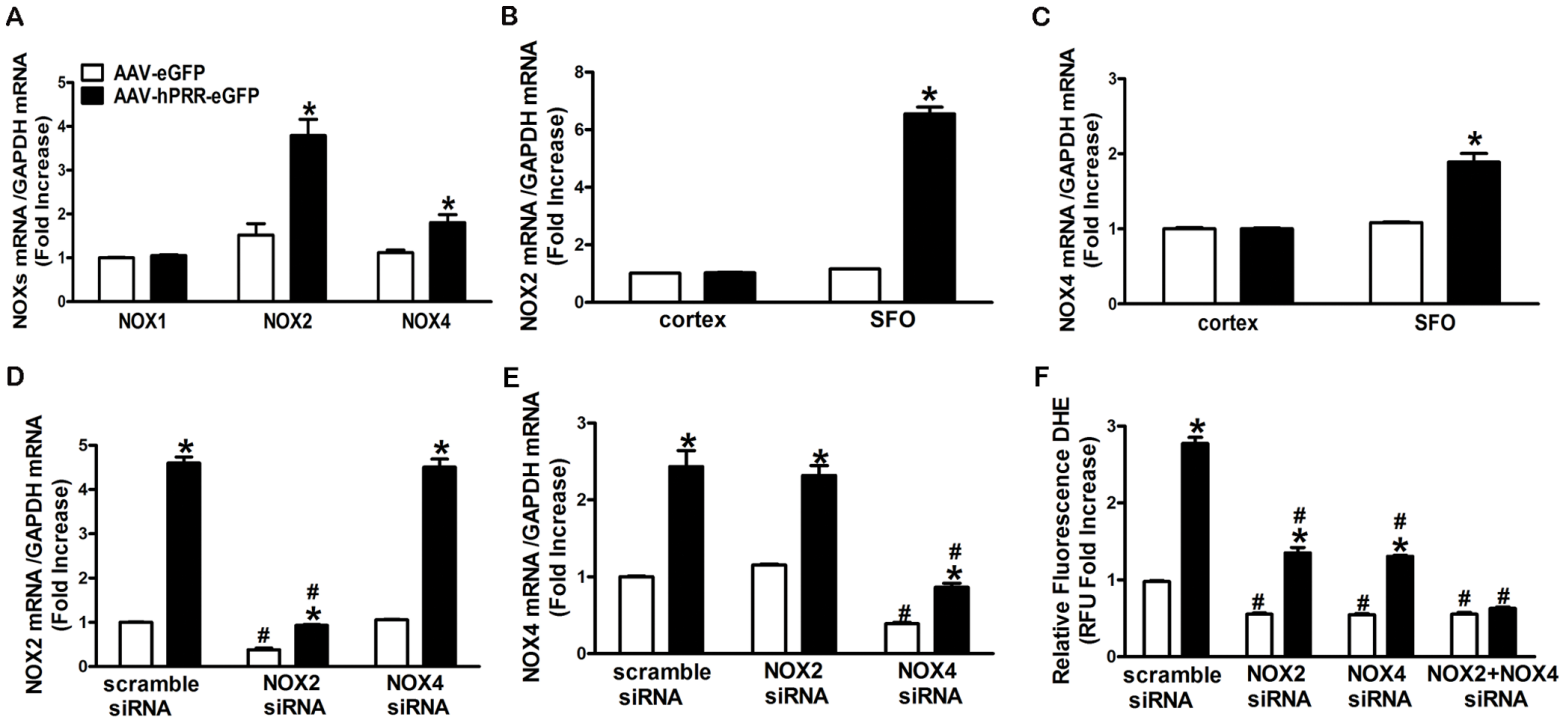
PRR mediates ROS production via up-regulation of NOX2 and NOX4 levels. NOX1, NOX2, and NOX4 mRNA levels in Neuro-2A cells (A). NOX2 (B) and NOX4 (C) mRNA levels in brain hypothalamic tissue 3 d after ICV injection of AAV-hPRR-eGFP or control virus. The knockdown efficiency of NOX2 siRNA (D) and NOX4 siRNA (E). A summary of the relative DHE fluorescence following NOX2 siRNA or NOX4 siRNA, or a combination of both (F). *P<0.05 vs. AAV-eGFP with scramble siRNA. # P<0.05 vs. scramble siRNA with the same virus.

We measured NOX1, NOX2, and NOX4 mRNA levels in Neuro-2A cells to determine whether NOX isoforms increased ROS production induced by human PRR over-expression. The PRR over-expression significantly increased both NOX2 and NOX4, but not NOX1 mRNA levels (Fig 4A, P<0.0001). We previously showed that ICV delivery of AAV induced a high level of protein expression in the SFO [17]. To confirm that this regulation also applies *in vivo*, we measured NOX2 and NOX4 mRNA levels in brain tissues after ICV administration of AAV-hPRR-eGFP or control virus for 72 h. The NOX2 and NOX4 mRNA expression levels were significantly increased in the SFO following PRR over-expression compared with controls (Fig 4B, C, P<0.001). The increase in the expression of the NOX isoforms was associated with the increase in human PRR expression levels in the SFO after ICV administration of AAV-hPRR-eGFP (Figure S2). In addition, we found that knock down of NOX2 and NOX4 expression using siRNA specifically inhibited NOX2 and NOX4 expression (70–80% inhibition) respectively (Figure 4D, E) without affecting the expression efficiency of endogenous mouse PRR and over-expression human PRR (Figure S3). Both NOX2 and NOX4 siRNA partially, but significantly, decreased the ROS production (Figure 4F) induced by human PRR over-expression and co-transfection of NOX2 and NOX4 siRNA completely reversed the PRR over-expression induced ROS formation (Figure 4F). Our results suggest that up-regulation of NOX2 and NOX4 mediates ROS production induced by human PRR over-expression.

### PRR Regulates NOX2 and NOX4 Expression via Activation of MAPK/ERK1/2 and PI3K/Akt Signaling Pathways

**Figure 5:**
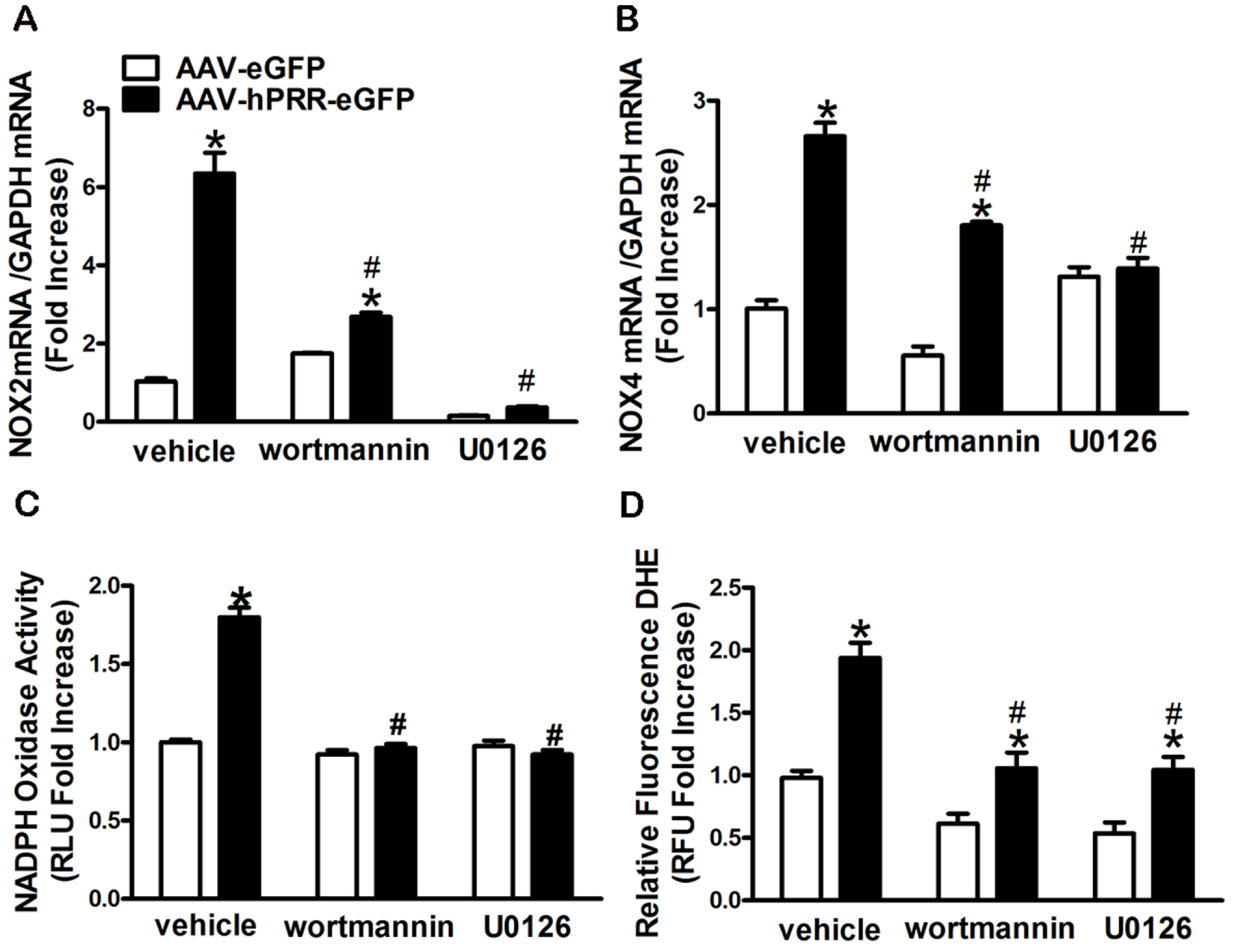
PI3K, MAPK activation mediates PRR over-expression-induced NOX2 and NOX4 up-regulation and ROS production.

**Figure 6:**
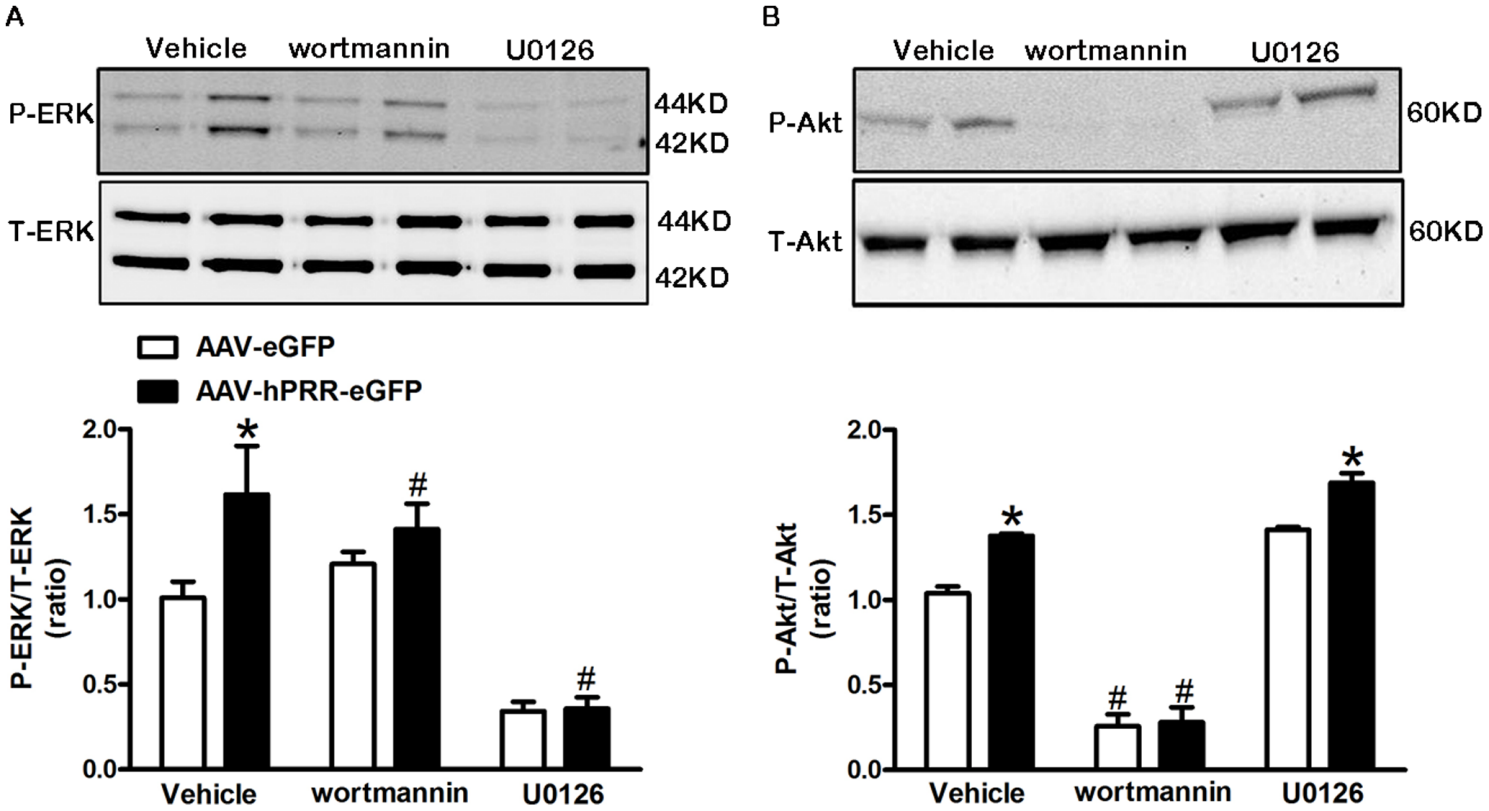
PRR over-expression activates ERK1/2 and Akt phosphorylation in Neuro-2A cells. A representative western blot and semi- quantitative graph for ERK1/2 (A) and Akt (B) phosphorylation in the presence of wortmannin (2 µM) or U0126 (10 µM) 3 d post AAV-hPRR-eGFP or control virus infection in Neuro-2A cells. All the experiments were done in the presence of losartan (10 µM). *P<0.05 vs. AAV-eGFP in the same treatment. # P<0.05 vs. vehicle treatment.

We found that inhibition of PI3K by wortmannin partially attenuated the PRR-induced NOX2 (Figure 5A) and NOX4 (Figure 5B) elevation; however, MAPK inhibition completely suppressed PRR-mediated NOX2 and NOX4 up-regulation (Figure 5A and B). Both PI3K and MAPK inhibition significantly reduced the NOX activity and ROS production (P<0.0001) induced by human PRR over-expression (Figure 5C, D). To elucidate the activation sequence of these molecules by the PRR, we measured ERK1/2 and Akt phosphorylation while one of the molecule’s activities was inhibited. The PI3K inhibition partially suppressed ERK1/2 phosphorylation (Figure 6A), whereas, inhibition of MAPK had no effect on PRR-induced Akt phosphorylation (Figure 6B) in Neuro-2A cells indicating that PI3K/Akt is up-stream of the MAPK/ERK1/2.

## Discussion

In this study, we found that human PRR over-expression increases ROS and superoxide production in neuronal cells via both Ang II-dependent and -independent pathways. We selected the Neuro-2A cell as our model because Neuro-2A cells contain all components of the RAS including angiotensinogen, renin, angiotensin converting enzyme 1 and 2, and the AT1R [18]–[20]. The Ang II-independent ROS formation appears to involve an activation of ERK1/2 and Akt, and up-regulation of NOX2 and NOX4 by the PRR. Moreover, prorenin mediates Ang II formation via binding to PRR in a dose-dependent manner. Prorenin mediated a significant increase in Ang II formation in Neuro-2A cells at a concentration of 2 nM or higher; however, when the prorenin concentration was lower than 2 nM, human PRR over-expression did not significantly alter Ang II levels. The rise in intracellular Ang II we observed may due to uptake from the cell surface and internalization via the AT1R or possibly intracellular formation of Ang II [21].

The K_D_ (8.3 nM) for rat prorenin to the human PRR is higher than that of human prorenin to the human PRR (1.2 nM) [22]. Although the K_D_ for mouse prorenin to human PRR has not been examined, in mouse neuronal cells, 2 nM of mouse prorenin is adequate to significantly elevate Ang II levels. Our data agrees with Biswas et al [22] showing that rodent prorenin exhibits activity to generate Ang II when binds to human PRR. However, studies from Kaneshiro et al. [23] contradict these findings. Whereas, studies from Danser’s group [24] indicate that an extremely high concentration of prorenin is required to form Ang II or initiate Ang II-independent MAPK activation via human PRR in isolated smooth muscle cells.

Due to the extremely low renin activity in the brain, debate has persisted regarding the existence of a functional brain RAS [25], [26]. In this study, we showed that prorenin levels were ten-fold higher than those of renin in the cell lysate. Furthermore, renin is undetectable in the cell culture medium indicating that renin might not be secreted into the extracellular space, at least in Neuro-2A cells, and the majority of the total (pro)renin was prorenin in the culture medium. The findings agree with previous reports that juxtaglomerular cells are the main source of renin as they have the machinery for processing and secreting renin; while other cell types do not [27]–[29]. Although, prorenin was previously recognized as a precursor of renin without aspartyl protease activity, prorenin can be activated when bound to the PRR, leading to the generation of Ang II [30]. The formation of Ang II from the binding of prorenin to the PRR in neuronal cells provides an alternative pathway for Ang II production without renin in the central nervous system.

**Figure 7:**
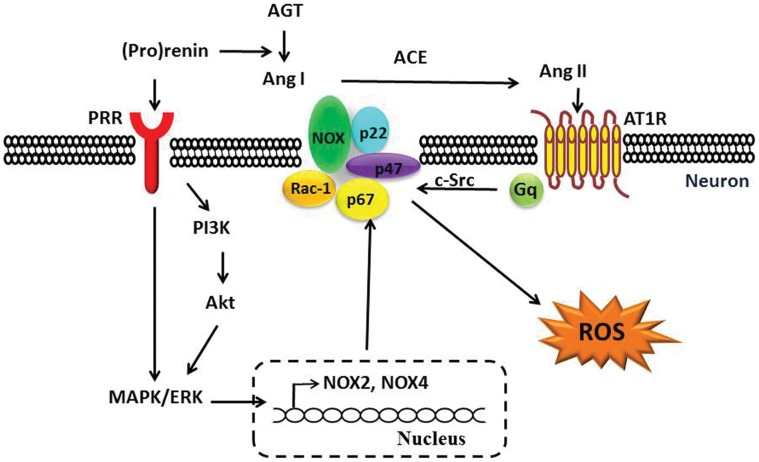
The Ang II-dependent and -independent PRR signals in ROS production. In neuronal cells, PRR over-expression activates Ang II-dependent and -independent ROS production via activation of PI3K/Akt and ERK1/2, up-regulation of NOX2 and NOX4, and subsequent increases in NADPH oxidase activity and ROS production, possibly through prorenin. Abbreviations: ROS, reactive oxygen species; PRR, (pro)renin receptor; AGT, angiotensinogen; Ang I, angiotensin I; ACE, angiotensin-converting enzyme; Ang II, angiotensin II; AT1R, angiotensin II type I receptor; NADPH oxidase, nicotinamide adenine dinucleotide phosphate-oxidase; PI3K, phosphoinositide-3-kinase; MAPK, mitogen activated protein kinase; ERK1/2, extracellular signal-regulated kinase.

In addition to Ang II-related functions, the PRR elicits Ang II-independent intracellular signals, including phosphorylation of PI3K/Akt [31] and activation of ERK1/2, up-regulation of transforming growth factor beta (TGF-β), plasminogen activator inhibitor (PAI-1), collagens, fibronectin [32], [33], and cyclooxygenase-2 [34]. Activation of PI3K/Akt [35] and MAPK [36] has been shown to up-regulate NOX4 gene expression. In addition, a recent study from Clavreul et al [37] found that the PRR mediates Ang II-independent NOX4 expression in embryonic kidney cells. Although the molecular mechanism by which this occurs was not addressed by the authors, their findings indicate a role of the PRR in regulating NOX4 expression. Our study showed that PRR over-expression regulates NOX2 and NOX4 expression through activation of Ang II-independent ERK1/2 and Akt phosphorylation in neuronal cells, and is linked to ROS production (Figure 7).

In summary, PRR over-expression mediates both Ang II-dependent and -independent ROS production in neuronal cells. The PRR-mediated Ang II-independent ROS production is, at least in part, via phosphorylation of ERK1/2 and PI3K/Akt, and up-regulation of NOX2 and NOX4 expression. We conclude that the PRR may mediate a new pathway for ROS production in the central nervous system.

## Supporting Information

Figure S1 **Ang II or PRR over-expression increases ROS production in neuronal cells.**
(DOCX)

Figure S2 **Mouse and human PRR mRNA expression in C57Bl/6J mice brain after ICV delivery of AAV.**
(DOCX)

Figure S3 **Mouse and human PRR mRNA expression levels in neuronal cells.**
(DOCX)
