# Supplementary material for: (Pro)renin Receptor Mediates Both Angiotensin II-Dependent and -Independent Oxidative Stress in Neuronal Cells
Source: PLoS One. 2013 Mar 14;8(3):e58339. doi: 10.1371/journal.pone.0058339 (PMC3597628; doi:10.1371/journal.pone.0058339)
Supplement: Figure S1 — Ang II or PRR over-expression increases ROS production in neuronal cells. (DOCX) [file pone.0058339.s001.docx]

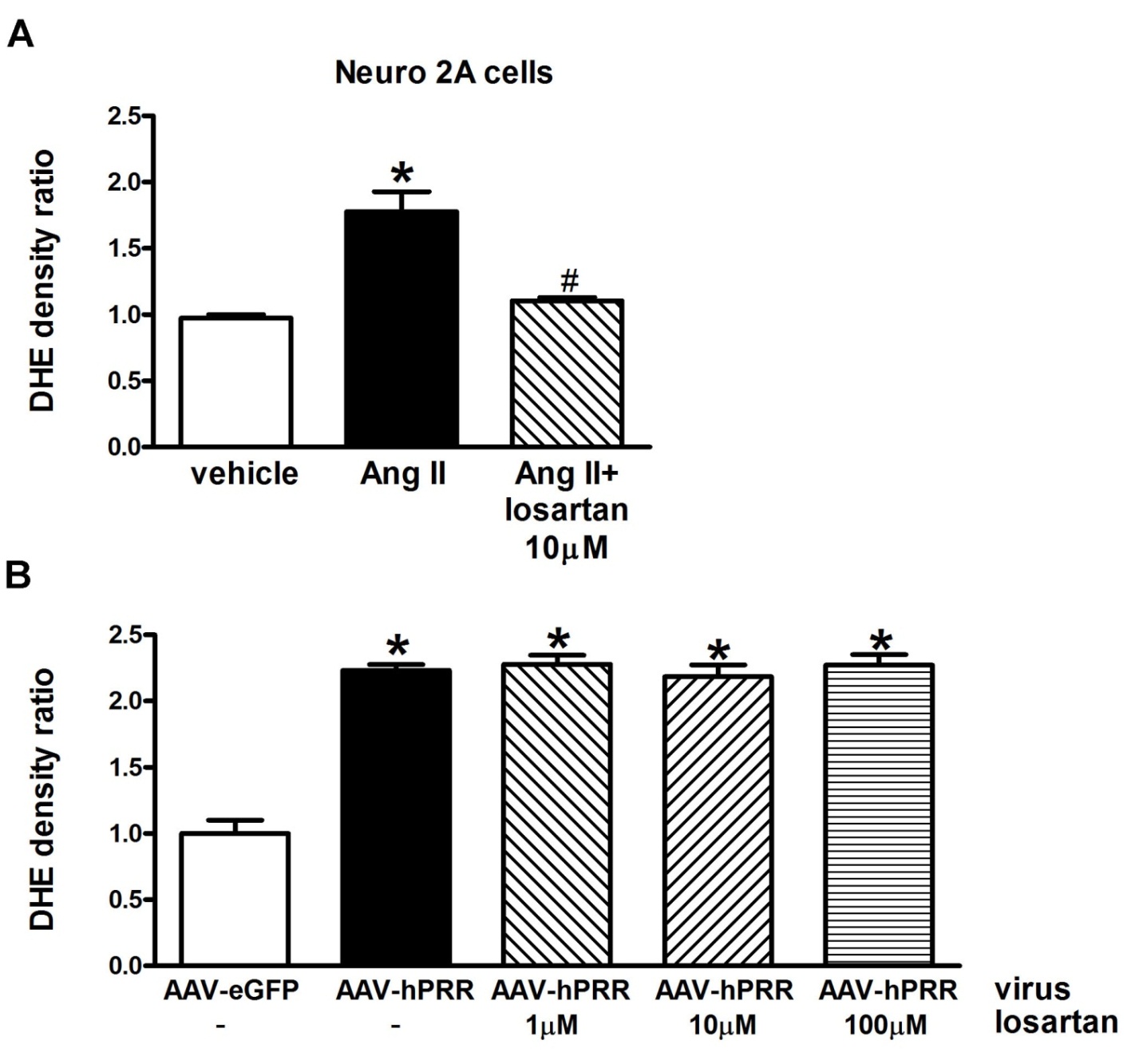


**Figure S1.** **Ang II or PRR over-expression increases ROS production in neuronal cells.**

(A) Neuro-2A cells were treated with Ang II (10 nM) for 20 min, with or without losartan (10 µM) for 30 min. * P<0.05 vs. vehicle, #<0.05 vs. Ang II. (B) Neuro-2A cells were infected with AAV-eGFP or AAV-hPRR-eGFP virus for three d, and incubated with different concentrations of losartan for 30 min. * P<0.05 vs. AAV-eGFP.
