# Supplementary material for: (Pro)renin Receptor Mediates Both Angiotensin II-Dependent and -Independent Oxidative Stress in Neuronal Cells
Source: PLoS One. 2013 Mar 14;8(3):e58339. doi: 10.1371/journal.pone.0058339 (PMC3597628; doi:10.1371/journal.pone.0058339)
Supplement: Figure S2 — Mouse and human PRR mRNA expression in C57Bl/6J mice brain after ICV delivery of AAV. (DOCX) [file pone.0058339.s002.docx]

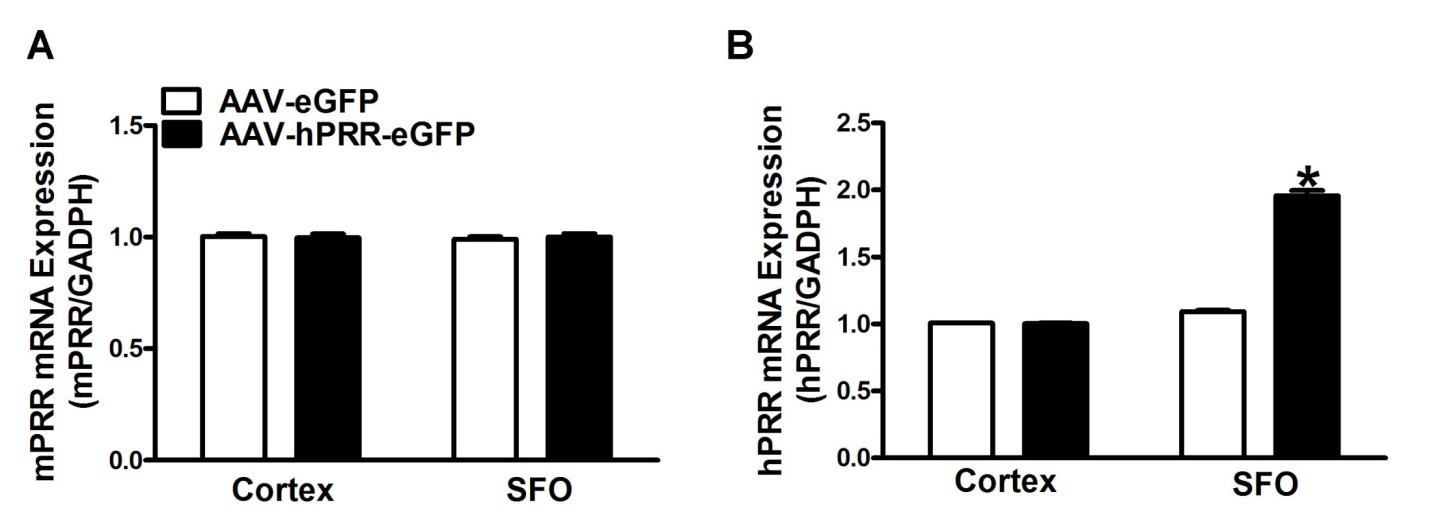


**Figure S2.** **Mouse and human PRR mRNA expression in C57Bl/6J mice brain after ICV delivery of AAV.**

Mice were injected with AAV-eGFP or AAV-hPRR-eGFP virus into the lateral ventricle of the brain. After 3 d, brain tissues were harvested for measurement of both mouse PRR mRNA and human PRR mRNA level in the SFO. Cortex tissues were included as control. * P<0.05 vs. AAV-eGFP.
