# Supplementary material for: (Pro)renin Receptor Mediates Both Angiotensin II-Dependent and -Independent Oxidative Stress in Neuronal Cells
Source: PLoS One. 2013 Mar 14;8(3):e58339. doi: 10.1371/journal.pone.0058339 (PMC3597628; doi:10.1371/journal.pone.0058339)
Supplement: Figure S3 — Mouse and human PRR mRNA expression levels in neuronal cells. (DOCX) [file pone.0058339.s003.docx]

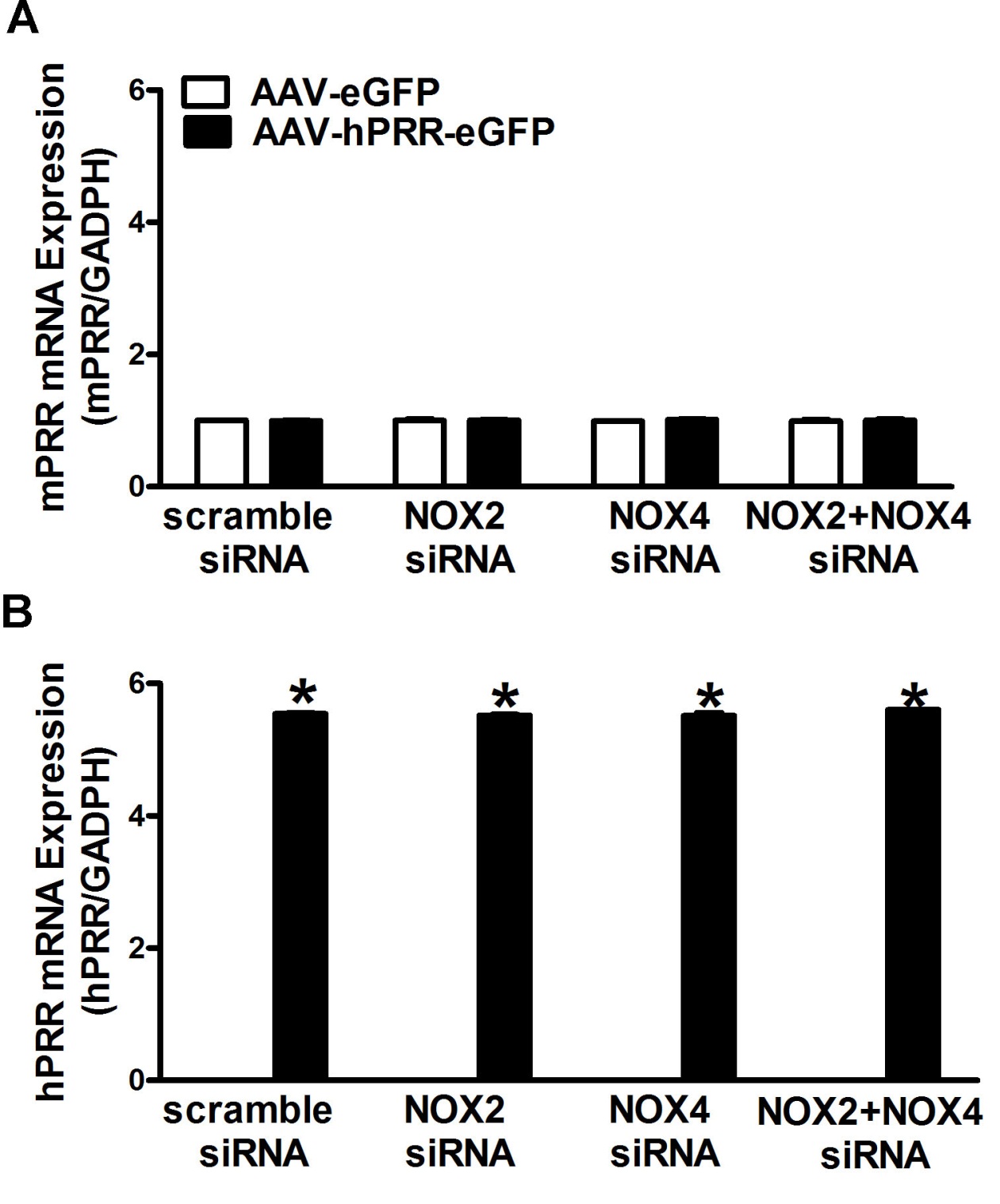


**Figure S3.** **Mouse and human PRR mRNA expression levels in neuronal cells.**

Neuro-2A cells were incubated with AAV-eGFP, AAV-hPRR-eGFP for two days. Cells were transfected with scramble siRNA, NOX2 siRNA, or NOX4 siRNA. After 48 h of transfection, mouse PRR (A) and human PRR (B) mRNA level were measured. * P<0.05 vs. AAV-eGFP.
